# Correction: Expanded graphite incorporated with Li_4_Ti_5_O_12_ nanoparticles as a high-rate lithium-ion battery anode

**DOI:** 10.1039/d4ra90055c

**Published:** 2024-05-15

**Authors:** Junkang Zhao, Xiayu Zhu, Wenfeng Zhang, Jingyi Qiu, Feiyue Zhai, Huimin Zhang, Gaoping Cao, Shengji Gao, Fei Ding, Yu Xiang

**Affiliations:** a Engineering and State Key Laboratory of Reliability and Intelligence of Electrical Equipment, School of Materials Science and Engineering, Hebei University of Technology Tianjin 300401 China hilldingfei@163.com; b Chemical Defense Institute, Academy of Military Sciences Beijing 100191 China drxiangyu2016@126.com; c Beijing Key Laboratory of Advanced Chemical Energy Storage Technology and Materials Beijing 100191 China; d Beihang University Beijing 100191 China

## Abstract

Correction for ‘Expanded graphite incorporated with Li_4_Ti_5_O_12_ nanoparticles as a high-rate lithium-ion battery anode’ by Junkang Zhao *et al.*, *RSC Adv.*, 2024, **14**, 11276–11283, https://doi.org/10.1039/D4RA00832D.

The authors regret that the one of the affiliations (affiliation *a*) was incorrectly shown in the original manuscript. The corrected affiliation list is as shown here.

The Royal Society of Chemistry apologises for these errors and any consequent inconvenience to authors and readers.

## Supplementary Material

